# Correlation Between *ALDH2* Gene Polymorphism and Coronary Artery Disease in Patients With Atrial Fibrillation

**DOI:** 10.1155/cdr/8733628

**Published:** 2026-01-15

**Authors:** Bo Zheng, Meiling Li, Peng Wang, Jialong Lin, Haifeng Pei, Hao Liu, Xianglin Ye

**Affiliations:** ^1^ Department of Neurology, Zigong Third People’s Hospital, Zigong, China, www.zgsyy.cn; ^2^ Department of Cardiology, The General Hospital of Western Theater Command, Chengdu, China; ^3^ Department of Emergency Surgery, Yancheng No.1 People′s Hospital, Affiliated Hospital of Medical School, Nanjing University, Yancheng, China, nju.edu.cn; ^4^ Department of Emergency Surgery, The First people′s Hospital of Yancheng, Yancheng, China; ^5^ College of Medicine, Southwest Jiaotong University, Chengdu, Sichuan, China, swjtu.edu.cn; ^6^ Department of Clinical Medicine, Southwest Medical University, Luzhou, China, swmu.edu.cn

**Keywords:** acetaldehyde dehydrogenase 2, atrial fibrillation, coronary artery disease, genotypic polymorphism, statins

## Abstract

**Objectives:**

The objectives of this study is to investigate the correlation between genotype polymorphism of aldehyde dehydrogenase 2 (ALDH2) and coronary artery disease (CAD) in atrial fibrillation patients.

**Methods:**

From November 2020 to December 2021, 80 patients with atrial fibrillation at a medical center in Chengdu were divided into two groups: CAD group (*n* = 25) and non‐CAD group (*n* = 55). The genotype composition ratio of ALDH2 (mutant‐type/wild‐type), blood biochemical indexes, the proportion of the history of lipid‐lowering drugs and time of taking lipid‐lowering drug were compared between the two groups.

**Results:**

The CAD group showed significantly lower levels of total cholesterol and low‐density lipoprotein cholesterol (LDL‐C) compared with the non‐CAD group (*p* < 0.05), and the proportion and time of taking lipid‐lowering drugs in CAD group were significantly increased (*p* < 0.05). The frequency of the ALDH2 genotype (mutant‐type/wild‐type) in the CAD group was notably elevated compared with that in the non‐CAD group (*p* < 0.05). In patients with atrial fibrillation, the risk of CAD in patients with ALDH2 mutant genotype (GA + AA) was 5.849 times that of ALDH2 wild‐type genotype (GG) (95*%*CI = 1.437–23.795, *p* < 0.05). The area under ROC curve of ALDH2 mutant genotype (GA + AA) was 0.624 (SE = 0.069, 95*%*CI : 0.488–0.759). In patients with ALDH2 wild‐type (GG), the levels of total cholesterol and LDL‐C in CAD group were significantly lower than those in non‐CAD group (*p* < 0.05). However, in patients with ALDH2 mutation genotype (GA + AA), the proportion of history of lipid‐lowering drugs in CAD group was significantly higher than that in non‐CAD group (*p* < 0.05).

**Conclusions:**

The polymorphism of ALDH2 gene is a high risk factor for CAD in patients with atrial fibrillation. The ALDH2 mutation genotype (GA + AA) may reduce the lipid‐lowering efficacy of statins.

## 1. Introduction

Aldehyde dehydrogenase 2 (ALDH2) is a crucial mitochondrial detoxification enzyme that plays a dual role in metabolizing harmful aldehydes, such as acetaldehyde and 2‐nonanal, into nontoxic carboxylic acids during ethanol metabolism. In addition to its detoxification function, ALDH2 mitigates oxidative damage and suppresses inflammatory responses in vascular endothelial cells, thereby contributing to the prevention and attenuation of cardiovascular pathologies, including coronary atherosclerosis [1, 2].

The ALDH2 gene is located on human chromosome 12q24.3 and encodes a polypeptide of 517 amino acids (58 kDa) that is predominantly expressed in the mitochondrial compartments of the liver, heart, kidney, and brain. A missense variant at the rs671 locus within exon 12 leads to the substitution of guanine (G) with adenine (A), resulting in a Glu504Lys substitution (p.Glu504Lys) in the structure of the ALDH2 protein. This genetic alteration compromises the catalytic efficiency of the enzyme, thereby diminishing its capacity for aldehyde detoxification [3]. The ALDH2 genetic polymorphism is primarily defined by the rs671 biallelic variant, resulting in two functionally distinct alleles: the wild‐type ALDH21 allele (retains catalytic activity) and the loss‐of‐function ALDH22 allele (catalytic activity abrogated). This variation determines enzyme functionality, with ALDH2∗2 carrying a complete loss of catalytic capacity due to the Glu504Lys substitution [4]. The ALDH2 locus exhibits three genotypic variants in human populations: GG (wild‐type), GA (heterozygous variant), and AA (homozygous variant). GG genotype can encode ALDH2 of normal activity level; while GA and AA genotypes are both mutant types, and the activity of ALDH2 encoded by GA is only 30%–40% of normal level, and the protein corresponding to AA type is almost inactive [5]. The ALDH2 rs671 variant demonstrates population‐specific prevalence, with the minor allele frequency reaching 30%–50% in East Asian populations. In contrast, this polymorphism exhibits negligible prevalence in non‐Asian populations [6]. Given the high prevalence of ALDH2 polymorphisms in Asian populations, investigating their pathophysiological correlations assumes particular significance. Notably, emerging evidence implies a cardioprotective role of ALDH2 in cardiovascular homeostasis, though the molecular mechanisms underpinning this association remain incompletely defined [7, 8]. However, the mechanism of action of ALDH2 in human body has not been fully elucidated. The relationship between ALDH2 gene polymorphism and coronary artery disease (CAD) is controversial [9, 10].

Atrial fibrillation (AF), the most prevalent arrhythmia, can significantly increase the risk of stroke and heart failure and pose a serious threat to human health. The incidence of AF has increased by more than four times over the past half century, and patients with AF are reported to be more likely to have lesions in their coronary arteries [11]. At present, there are more than 46 million patients with AF in the world [12]. Epidemiological data indicate that AF is associated with a five‐ to six‐fold increased risk of embolic stroke compared with age‐matched controls [13]. AF pathogenesis is influenced by multiple risk factors, including age, gender, smoking, alcohol consumption, and obesity. Oxidative stress‐mediated accumulation of reactive oxygen species and toxic aldehydes has emerged as a key pathogenic mechanism in AF [14]. Therefore, ALDH2 associated with toxic aldehyde degradation can bring benefits to patients with AF in the direction of reducing oxidative stress. Animal studies have demonstrated that ALDH2 may participate in the cardioprotective effect of sphingosine‐1 phosphate‐associated anti‐renin–angiotensin system activation [15]. However, the relevant clinical trial conclusion focused more on the relationship between alcohol consumption habit change of ALDH2 variant A allele carriers and AF. A study conducted by Yamashita et al. [16] involving 656 patients with AF showed that the ALDH2 mutant genotype GA was not associated with AF. Prolonged alcohol exposure may induce AF in GA genotype carriers through impaired ethanol metabolism. In contrast, carriers of the AA genotype exhibited a reduced likelihood of sustained alcohol consumption, attributed to significantly diminished ALDH2 activity and ethanol metabolism rates, which correlate with a lower prevalence of AF compared with GA carriers. These findings underscore the necessity for further exploration of ALDH2 genotype‐specific mechanisms underlying AF pathogenesis. To date, no studies have investigated the association between ALDH2 genetic variants and CAD in patients with AF. This study enrolled patients with AF to evaluate the interactions between ALDH2 polymorphisms and CAD, with the goal of identifying novel genetic‐based strategies for the prevention and treatment of cardiovascular disease.

## 2. Materials and Methods

### 2.1. Research Objects

**Figure 1:**
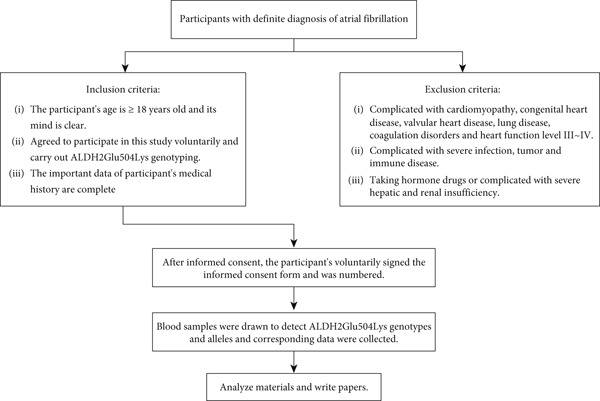
Test flow chart.

From November 2020 to December 2021, a total of 80 patients with AF were enrolled at a medical center in Chengdu, based on the order of their diagnosis. Among these, 25 patients had CAD, while 55 patients did not (Figure 1). The inclusion criteria were as follows: (1) participants must be aged 18 years or older and possess clear cognitive function; (2) participants must voluntarily agree to partake in this study and provide consent for ALDH2 Glu504Lys genetic testing; (3) participants must have complete and significant medical history data. The exclusion criteria included: (1) patients with complications such as cardiomyopathy, congenital heart disease, valvular heart disease, lung disease, coagulation disorders, or heart function classified as Level III to IV; (2) patients with severe infections, tumors, or immune diseases; (3) patients who are on hormone therapy or have severe hepatic and renal insufficiency. The diagnosis of AF and the classification criteria for paroxysmal and persistent AF were in accordance with *2023 ACC/AHA/ACCP/HRS Guideline for the Diagnosis and Management of Atrial Fibrillation* [17], and the degree of stenosis of single or multiple coronary arteries ≥ 50% in coronary angiography results was the diagnostic criterion of CAD. The genotype data were collected from peripheral venous blood specimens (2–3 mL of peripheral venous blood shaken slowly and stored at −80°C) using the ALDH2 (Glu504Lys) gene kit (Shanghai BaiOTech Co., Ltd.). Alcohol consumption [18]: individuals with ≥ 250 mL/week of 42% (alcohol by volume) alcohol consumption for ≥ 2 years were categorized as having prolonged alcohol exposure, while nondrinkers and those below these thresholds were classified as nonexposed. All of the lipid‐lowering drugs taken by patients included were statins (atorvastatin or rosuvastatin). This study was approved by the Ethics Committee of the General Hospital of Western Theater Command (2021EC5‐116).

### 2.2. Statistical Methods

SPSS software (SPSS 26.0) was used for the statistical analysis. The measurement data underwent testing for normal distribution and equality of variance. The measurement data following the normal distribution was represented by the mean ± standard deviation ($\overset{®}{\text{x}}\pm \text{SD}$) and compared with T test or single factor ANOVA test. The measurement data not meeting the normal distribution were taken as the median M (P25, P75) and nonparametric rank sum test or Kruskal–Wallis test was used for analysis. The counting data were presented as [*n* (%)], and the Chi‐square test was utilized to compare the counting data across different groups. Logistic regression analysis and ROC curve were used to analyze the association of occurrence of CAD in patients with AF with ALDH2 Glu504Lys genotype. A chi‐square test was used to check compliance with Hardy–Weinberg equilibrium after determining allele frequencies in gene counts. *p* < 0.05 presents statistically significant difference.

## 3. Results

### 3.1. Characterization of ALDH2 Gene in Patients With AF

The ALDH2 Glu504Lys genotype in patients with AF was classified into three categories: GG wild genotype, GA mutant genotype, and AA mutant genotype. Among the 80 patients analyzed, 52 exhibited the GG wild genotype (65.00%), which included 12 patients with CAD and 40 patients without CAD. In contrast, 28 patients presented with either the GA or AA mutation, accounting for 35.00% of the cohort. Specifically, there were 25 cases of GA mutation (31.25%), consisting of 10 patients with CAD and 15 without. Additionally, three patients had the AA mutation genotype, representing 3.75% of the total; all of these patients had CAD. The G allele of ALDH2 Glu504Lys was the predominant allele, comprising 80.6% of the alleles, while the A allele accounted for 19.4%. The Hardy–Weinberg equilibrium test indicated that the collected samples were representative of the population (*p* > 0.05), as illustrated in Table 1.

**Table 1:** Hardy–Weinberg equilibrium test for ALDH2 Glu504Lys genotype in patients with AF.

	**Genotype (actual frequency [theoretical frequency])**			**Allelic gene (actual frequency [frequency])**		**X** ^2^	**p** **value**
	**GG**	**GA**	**AA**	**G**	**A**		
Non‐ CAD	40 (41.02)	15 (12.95)	0 (1.02)	95 (0.864)	15 (0.136)	1.371	0.504
CAD	12 (11.56)	10 (10.88)	3 (2.56)	34 (0.680)	16 (0.320)	0.164	0.921
Total	52 (52.00)	25 (24.99)	3 (3.00)	129 (0.806)	31 (0.194)	< 0.001	1.000

Abbreviation: CAD: coronary artery disease.

### 3.2. Basic Data Analysis of Patients With AF

Patients with AF were classified into two groups: the noncoronary artery disease (non‐CAD) group (*n* = 55) and the CAD group (*n* = 25). The CAD group demonstrated significantly lower serum levels of total cholesterol and LDL‐C compared with the non‐CAD group (*p* < 0.05). In contrast, the CAD group showed a significant increase in age, the proportion of the ALDH2 genotype (GA + AA/GG), the history of lipid‐lowering drug use, and the duration of lipid‐lowering drug treatment compared with the non‐CAD group (*p* < 0.05), as detailed in Table 2.

**Table 2:** Comparison of general clinical data between patients with AF in non‐CAD group and those in CAD group.

**Item**	**Non-CAD group (** **n** = 55**)**	**CAD group (** **n** = 25**)**	**T/X2/Z**	**p** **value**
Male (cases [%])	33 (60.000)	18 (72.000)	1.071	0.301
History of persistent AF (cases [%])	32 (58.182)	16 (64.000)	0.242	0.622
History of hypertension (cases [%])	24 (43.636)	15 (60.000)	1.842	0.175
History of drinking (cases [%])	10 (18.182)	7 (28.000)	0.990	0.320
BMI (kg/m^2^)	24.181 ± 3.251	24.458 ± 3.431	−0.347	0.729
Creatinine (*μ*mol/L)	75.000 (66.000, 87.000)	82.000 (70.500, 102.500)	−1.641	0.101
Glutamic oxaloacetic transaminase (IU/L)	23.200 (18.600, 30.800)	24.100 (18.750, 28.950)	−0.176	0.860
hs‐CRP (mg/L)	1.180 (0.680, 3.160)	0.900 (0.535, 1.980)	−0.836	0.403
Troponin (*μ*g/L)	0.007 (0.001, 0.022)	0.010 (0.005, 0.030)	−1.800	0.072
BNP (pg/L)	163.380 (77.080, 300.220)	230.470 (133.295, 507.420)	−1.583	0.113
EF (%)	59.000 (53.000, 61.000)	53.000 (48.500, 61.000)	−1.436	0.151
HDL‐C (mmol/L)	1.230 (1.030, 1.510)	1.270 (0.960, 1.370)	−0.696	0.487
Triglyceride (mmol/L)	1.230 (1.010, 1.700)	1.060 (0.950, 1.400)	−1.853	0.064
Uric acid (*μ*mol/L)	390.000 (333.000, 442.000)	387.000 (360.500, 432.500)	−0.415	0.678
Internal diameter of left atrium (mm)	42.000 (37.000, 47.000)	47.000 (38.000, 54.000)	−1.954	0.051
ALDH2 genotype ([GA + AA]/GG [case])	15/40	13/12	4.619	0.032 ^∗^
Age (years)	66.000 (57.000, 74.000)	75.000 (69.000, 78.000)	−3.071	0.002 ^∗∗^
Total cholesterol (mmol/L)	4.210 (3.510, 4.840)	3.000 (2.620, 4.270)	−3.207	0.001 ^∗∗^
LDL‐C (mmol/L)	2.530 (2.060, 3.020)	1.730 (1.290, 2.600)	−3.498	< 0.001 ^∗∗^
Time of taking lipid‐lowering drugs (months)	16.000 (0, 22.000)	24.000 (16.000, 26.000)	−3.199	0.001 ^∗∗^
History of lipid‐lowering drugs (cases [%])	28 (50.909)	22 (88.000)	10.089	0.001 ^∗∗^

Abbreviation: AF: atrial fibrillation; BMI: body mass index; BNP: brain natriuretic peptide; EF: ejection fraction; HDL‐C: high density lipoprotein cholesterol; hs‐CRP: hypersensitive C reactive protein; LDL‐C: low density lipoprotein cholesterol.

^∗^ *p* < 0.05.

^∗∗^ *p* < 0.01.

### 3.3. Analysis of Risk Factors of CAD in Patients With AF

In Table 3, univariate logistic regression analysis identified the ALDH2 Glu504Lys (GA + AA) genotype and age as significant risk factors for CAD in patients with AF (*p* < 0.05). After adjusting for BMI, hs‐CRP, and serum glutamic oxaloacetic transaminase levels, multivariable analysis confirmed that the ALDH2 Glu504Lys (GA + AA) genotype, age, and LDL‐C were independent correlates of CAD (*p* < 0.05). Compared with carriers of the GG genotype, subjects with the GA + AA genotype exhibited a 5.849‐fold increased risk of CAD (95% confidence interval [CI] 1.437–23.795, *p* = 0.014). These results indicate that the polymorphism of the ALDH2 gene is a strongly associated factor for CAD in patients with AF.

**Table 3:** Logistic regression analysis of ALDH2 Glu504Lys genotype and CAD risk in patients with AF.

**Variable**	***β* value**	**SE**	**Wald**	**OR (95% CI)**	**p** **value**
Univariate analysis					
History of drinking	−0.206	1.158	0.032	0.814 (0.084~7.867)	0.859
Creatinine (*μ*mol/L)	0.004	0.012	0.103	1.004 (0.98~1.028)	0.748
Glutamic oxaloacetic transaminase (IU/L)	−0.086	0.053	2.585	0.918 (0.827~1.019)	0.108
hs‐CRP (mg/L)	−0.074	0.039	3.572	0.928 (0.859~1.003)	0.059
Troponin (*μ*g/L)	3.929	3.896	1.017	50.878 (0.025~105309.176)	0.313
BNP (pg/L)	0.001	0.002	0.159	1.001 (0.997~1.004)	0.690
EF (%)	−1.769	5.380	0.108	0.171 (0 ~ 6475.765)	0.742
Male	1.564	1.236	1.603	4.780 (0.424~53.862)	0.206
Total cholesterol (mmol/L)	2.208	1.846	1.430	9.095 (0.244~338.989)	0.232
Triglyceride (mmol/L)	−0.184	0.688	0.071	0.832 (0.216~3.204)	0.790
LDL‐C (mmol/L)	−4.558	2.358	3.738	0.010 (0.000~1.065)	0.053
HDL‐C (mmol/L)	−2.456	3.351	0.537	0.086 (0.000~61.032)	0.464
Uric acid (*μ*mol/L)	0.000	0.004	0.000	1.000 (0.992~1.008)	0.994
Internal diameter of left atrium (mm)	−0.078	0.061	1.674	0.925 (0.821~1.041)	0.196
History of hypertension	−0.544	0.900	0.365	0.581 (0.100~3.387)	0.546
History of persistent AF	0.468	0.929	0.254	1.597 (0.259~9.859)	0.614
BMI (kg/m^2^)	0.268	0.147	3.325	1.307 (0.980~1.742)	0.068
GA + AA genotype	2.001	0.877	5.202	7.396 (1.325~41.283)	0.023 ^∗^
Age (years)	0.120	0.057	4.34	1.127 (1.007~1.261)	0.037 ^∗^
Multivariate analysis					
hs‐CRP (mg/L)	−0.067	0.037	3.285	0.936 (0.87~1.005)	0.070
BMI (kg/m^2^)	0.138	0.095	2.133	1.148 (0.954~1.383)	0.144
Glutamic oxaloacetic transaminase (IU/L)	−0.056	0.030	3.435	0.945 (0.891~1.003)	0.064
Age (years)	0.088	0.039	4.943	1.092 (1.010~1.179)	0.026 ^∗^
GA + AA genotype	1.766	0.716	6.085	5.849 (1.437~23.795)	0.014 ^∗^
LDL‐C (mmol/L)	−1.555	0.491	10.054	0.211 (0.081~0.552)	0.002 ^∗∗^

Abbreviations: BMI: body mass index; BNP: brain natriuretic peptide; EF: ejection fraction; HDL‐C: high density lipoprotein cholesterol; hs‐CRP: hypersensitive C recreative protein; LDL‐C: low density lipoprotein cholesterol.

^∗^ *p* < 0.05.

^∗∗^ *p* < 0.01.

### 3.4. ROC Curve Results Analysis

**Figure 2:**
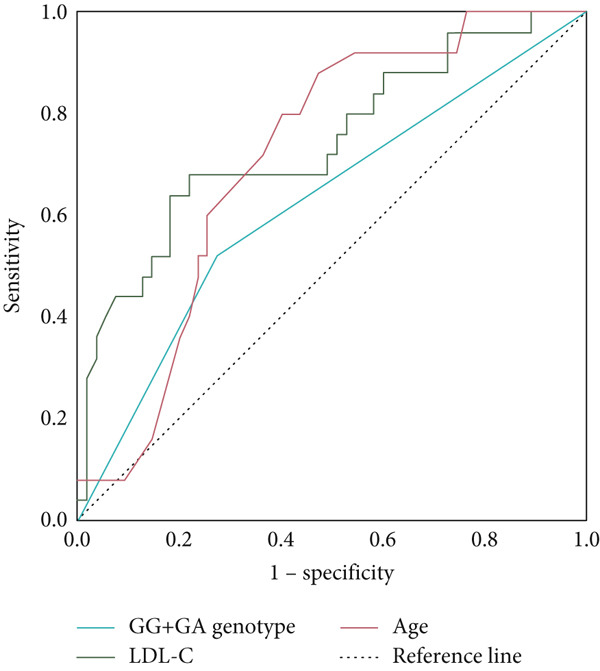
ROC curve of association between age, LDL‐C, and ALDH2 mutation genotype (GA + AA) and risk of CAD in patients with AF.

ROC curve analysis based on multivariable logistic regression was conducted to evaluate the predictive utility of age, LDL‐C, and the ALDH2 GA + AA genotype for CAD in patients with AF, as illustrated in Figure 2. The GA + AA genotype exhibited a moderate predictive capacity for CAD, with an AUC of 0.624 (standard error [SE] = 0.069, 95% CI: 0.488–0.759), while age demonstrated a higher AUC of 0.715 (SE = 0.058, 95% CI: 0.602–0.828). LDL‐C presented an AUC of 0.745 (SE = 0.062, 95% CI: 0.133–0.377). Collectively, these findings underscore the ALDH2 GA + AA genotype as a significant risk factor for CAD in AF populations.

### 3.5. Correlation Analysis Between ALDH2 Genotype and Blood Lipid Level/Lipid‐Lowering Drug Efficacy in Patients With AF

Patients were stratified into ALDH2 wild‐type (GG) and mutant (GA + AA) groups, which were further categorized based on CAD status. Comparisons across the four subgroups focused on triglyceride levels, high‐density lipoprotein cholesterol (HDL‐C), total cholesterol, and LDL‐C, as well as alcohol consumption history and the utilization of lipid‐lowering therapy (both proportion and duration). Wild‐type (GG) patients in the non‐CAD subgroup (*n* = 40) exhibited significantly higher total cholesterol and LDL‐C levels compared with patients with GG‐CAD (*n* = 12) (*p* < 0.05). In contrast, no significant differences in these lipid parameters were observed between the GA + AA non‐CAD (*n* = 15) and GA + AA CAD (*n* = 13) subgroups (*p* > 0.05). Notably, GA + AA non‐CAD patients demonstrated a significantly lower proportion of lipid‐lowering therapy users compared with GA + AA CAD patients (*p* < 0.05), despite having equivalent lipid profiles. Other variables, including triglycerides, HDL‐C, and alcohol consumption history, showed no significant intergroup differences (*p* > 0.05). The duration of lipid‐lowering therapy also revealed no statistically significant differences across the four subgroups (*p* > 0.05). Our results suggest that ALDH2 genetic polymorphism may impair the efficacy of statin‐induced lipid lowering, as indicated by equivalent lipid parameters despite inconsistent therapy utilization (Table 4).

**Table 4:** Comparison of data on blood lipids, history of drinking, and lipid‐lowering drug use in patients with AF.

	**GG genotype (** **n** = 52**)**		**(GA + AA) genotype (** **n** = 28**)**		**p** **value** ^**a**^	**p** **value** ^**b**^	**p** **value** ^**c**^	**p** **value** ^**d**^	**p** **value** ^**e**^
	**Non-CAD group (** **n** = 40**)**	**CAD group (** **n** = 12**)**	**Non-CAD group (** **n** = 15**)**	**CAD group (** **n** = 13**)**					
Triglyceride (mmol/L)	1.180 (1.013, 1.700)	1.095 (1.013, 1.590)	1.370 (0.920, 2.040)	1.050 (0.860, 1.325)	—				0.281
HDL‐C (mmol/L)	1.255 ± 0.317	1.100 ± 0.263	1.261 ± 0.265	1.262 ± 0.296	0.109	0.993	0.946	0.168	0.396
History of drinking (cases [%])	8 (20.000)	5 (41.667)	2 (13.333)	2 (15.385)	0.147	1.000	0.710	0.202	0.323
Time of taking lipid‐lowering drugs (months)	17.000 (0.000, 22.000)	23.000 (16.500, 25.750)	0.000 (0.000, 26.000)	25.000 (16.000, 26.000)	0.217	0.140	1.000	1.000	0.016 ^∗^
Total cholesterol (mmol/L)	4.23 (3.655, 4.900)	2.865 (2.478, 3.948)	4.010 (3.200, 4.810)	3.250 (2.690, 4.405)	0.018 ^∗^	1.000	1.000	1.000	0.010 ^∗^
LDL‐C (mmol/L)	2.585 (2.073, 3.055)	1.415 (1.323, 2.423)	2.320 (1.630, 3.020)	1.840 (1.215, 2.750)	0.009 ^∗∗^	0.743	1.000	1.000	0.004 ^∗∗^
History of lipid‐lowering drugs (cases [%])	22 (55.000)	10 (83.333)	6 (40.000)	12 (92.308)	0.099	0.006 ^∗∗^	0.322	0.593	0.005 ^∗∗^

*Note:* The total cholesterol, triglyceride, LDL‐C and the time of taking lipid‐lowering drugs did not meet the normal distribution. Kruskal–Wallis test was used. The *p* value between the two groups was adjusted by Bonferroni correction. HDL‐C met the normal distribution, and single factor ANOVA test was adopted. The *p* value between the two groups was obtained by LSD method. The chi‐square test of R × C contingency table was used to compare the history of drinking and the history of taking lipid‐lowering drugs. *p* values between the two groups were obtained by chi‐square test of four‐fold table.

Abbreviations: HDL‐C: High‐density lipoprotein cholesterol; LDL‐C: low‐density lipoprotein cholesterol.

^a^In GG genotype patients, the non‐CAD group was compared with the CAD group.

^b^In (GA + AA) genotype patients, the non‐CAD group was compared with the CAD group.

^c^Comparison between GG and (GA + AA) genotypes in non‐CAD group.

^d^Comparison between GG and (GA + AA) genotypes in CAD group.

^e^Comparison among the four groups.

^∗^ *p* < 0.05.

^∗∗^ *p* < 0.01.

## 4. Discussion

In recent years, the incidence of CAD has increased from 27.3% to 31.4%. For patients with AF, the probability of coronary artery lesion is higher [19]. Epidemiological data indicate that patients with AF exhibit a twofold increased risk of myocardial infarction compared to age‐matched controls [20]. At the same time, studies have proved that ALDH2 can protect the heart. Adeniji et al. [8] found that elevated ALDH2 expression exerted significant cardioprotective effects by suppressing atherosclerosis and myocardial cell apoptosis. Another investigations demonstrate that ALDH2 can improve mitochondrial respiratory efficiency and suppress mitochondrial permeability transition and cytochrome c release induced by calcium ions in failing heart [7], thereby improving the function of myocardial mitochondria. The ALDH2 polymorphism, particularly the ∗2 allele (rs671, Glu504Lys), has emerged as a critical genetic factor influencing the clinical and translational aspects of CAD, especially in high‐prevalence regions such as East Asia and other populations with significant carrier frequencies [21, 22]. This polymorphism, which reduces ALDH2 enzymatic activity to 1%–6% of the wild‐type, is associated with a spectrum of cardiovascular pathologies, including ACS, MI, and atherosclerosis [2, 23]. The two allele’s prevalence in East Asians, affecting approximately 40% of the population, underscores its substantial public health impact [24]. The translational implications of ALDH2 polymorphism are profound, particularly in the context of precision medicine. For instance, ALDH2∗2 carriers exhibit altered responses to nitroglycerin, a common treatment for angina, because of impaired conversion of nitroglycerin to nitric oxide, thereby reducing its therapeutic efficacy [25]. This highlights the need for genotype‐specific therapeutic strategies, such as the use of ALDH2 activators like Alda‐1 and Alda‐64, which have shown promise in preclinical models by restoring enzymatic activity and mitigating oxidative stress [26, 27]. Additionally, ALDH2 polymorphism has been linked to increased susceptibility to myocardial ischemia/reperfusion (I/R) injury, a major contributor to adverse outcomes in patients with ACS [28]. Experimental studies have demonstrated that ALDH2 overexpression attenuates I/R injury by reducing 4‐HNE accumulation and preserving mitochondrial function, suggesting that enhancing ALDH2 activity could be a viable therapeutic approach [29]. Epidemiological evidence further underscores the clinical relevance of ALDH2 polymorphism in CAD. Meta‐analyses have consistently shown that ALDH2∗2 carriers have a higher risk of MI and ACS, with odds ratios significantly elevated in East Asian populations [30, 31]. Moreover, ALDH2 polymorphism has been implicated in the pathogenesis of coronary spasm, a condition prevalent in Japanese patients with ST‐segment elevation myocardial infarction (STEMI), further highlighting its role in diverse CAD phenotypes [32].

In this research, multivariable logistic regression analysis indicated that patients with AF carrying the ALDH2 variant genotypes (GA/AA) exhibited a 5.849‐fold increased risk of developing CAD compared with patients with AF with the wild‐type GG genotype. Furthermore, there was a significantly higher proportion of patients with both AF and CAD who possessed the GA + AA/GG genotypes compared with those with AF alone. The mechanism by which ALDH2 polymorphism influences the risk of CAD in patients with AF may be attributed to several factors: (1) the decreased enzyme activity associated with the GA and AA genotypes reduces the degradation rate of toxic aldehydes, leading to an increased formation of reactive oxygen species that can damage cardiomyocytes [33]; (2) reduced enzymatic activity associated with GA/AA genotypes was linked to enhanced vascular endothelial inflammation, thereby increasing susceptibility to atherosclerosis and contributing to the pathogenesis and progression of CAD; (3) the mutant ALDH2 allele can enhance glycolysis and pentose phosphate pathway, thereby disrupting cardiac lipid and glucose metabolism [34], promote myocardial fibrosis and atrial remodeling, and in turn cause the occurrence and progression of AF. The irregular atrial contraction and rapid ventricular rate caused by AF can increase cardiac oxygen consumption while reducing cardiac output, resulting in imbalance of blood supply, oxygen consumption, and thrombosis [19]. These alternations result in impaired cardiac systolic and diastolic function and worsening myocardial ischemia, thereby synergizing with oxidative stress to accelerate CAD progression.

In parallel, logistic regression analysis and ROC curve showed that increasing age significantly increased the risk of CAD in patients with AF, consistent with previous reports [35]. In this study, the total cholesterol and LDL‐C levels in patients with AF and CAD were found to be lower than those in patients without CAD. This finding is primarily attributed to the administration of statins following the diagnosis of CAD. Lipid‐lowering agents, particularly statins, are fundamental in the management of CAD, demonstrating substantial effects on enhancing cardiovascular outcomes through their lipid‐lowering mechanisms. These agents contribute to the improvement of the coronary condition in patients with CAD by decreasing blood lipid levels and mitigating vascular inflammation [36]. In this study, we collected data on lipid‐lowering agent exposure duration and analyzed their associations with ALDH2 genotypes and CAD status. Notably, among patients with AF carrying the wild‐type (GG) allele, total cholesterol, and LDL‐C levels were significantly elevated in non‐CAD subjects compared with patients with CAD (*p* < 0.05). In contrast, no such difference was observed in total cholesterol and LDL‐C levels between non‐CAD and CAD groups among patients with the ALDH2 variant genotype (GA + AA) (*p* > 0.05). This discrepancy may reflect the protective role of the ALDH2 variant genotype through modulating lipid metabolism‐related enzymatic activity. Animal studies by Zhong et al. [37] shown that ALDH2 mutation or ALDH2 deficiency can stabilize the key enzyme by cholesterol biosynthesis, HMG‐CoA reductase, leading to an increase in cholesterol synthesis, but this effect can be inhibited by lovastatin. While our current findings do not demonstrate this effect, this discrepancy may be attributed to multiple factors, including interspecies differences (human vs. animal models), pharmacological formulation variations of lipid‐lowering agents, and sample size limitations. Meanwhile, the above study also showed that under the presence of excess cholesterol, ALDH2 may reduce cholesterol synthesis by potentiating ubiquitination and degradation of HMG‐CoA reductase. LDL‐C of human body is mainly composed of phospholipids, cholesterol, apolipoprotein B100, and triglyceride. The apolipoprotein B100 can recognize and bind to the low‐density lipoprotein receptor on the cell membrane and make LDL‐C enter the cell via endocytosis, and is decomposed into free cholesterol by the action of lysosomes. Luo et al. [38] showed that the ALDH2 mutant genotype may reduce lysosomal hydrogen pump expression, thereby reducing lipolysis of lipoproteins, and inhibiting the efficacy of statins in reducing LDL‐C. However, the changes of exercise and diet in patients with CAD may also promote the result that LDL‐C and total cholesterol in patients without CAD are significantly higher than those in patients with CAD.

Based on our findings, intensified lipid management in patients with AF may represent a potential strategy for the prevention of CAD. However, several limitations of our study warrant consideration. Firstly, this investigation is an observational, single‐center study with a limited sample size, and its conclusions require validation through prospective multicenter trials involving larger and more diverse populations. Secondly, the precise molecular mechanisms underlying the effects of ALDH2 polymorphism on lipid homeostasis remain to be elucidated, necessitating further exploration. In summary, this study indicates that ALDH2 gene polymorphisms may be a significant risk factor for CAD in patients with AF, and ALDH2 gene mutations (GA + AA) may diminish the lipid‐lowering efficacy of statins. Our study provides a novel perspective for the prevention and treatment of clinical cardiovascular diseases; however, the specific mechanisms by which it influences the pathogenesis of CAD require further investigation.

## Ethics Statement

This study was approved by the Ethics Committee of the General Hospital of Western Theater Command (2021EC5‐116).

## Consent

Informed consent was obtained from all subjects involved in the study.

## Conflicts of Interest

The authors declare no conflicts of interest.

## Author Contributions

Bo Zheng: idea support, data collecting and analysis, paper writing; Meiling Li: data collecting and analysis, paper writing; Peng Wang: idea support, data collecting, paper writing; Jialong Lin: idea support, data collecting; Xianglin Ye: idea support, paper editing; Hao Liu: data analysis, paper writing, paper correcting; Haifeng Pei: idea support, paper correcting, funding.

## Funding

This study was supported by Noncommunicable Chronic Diseases‐National Science and Technology Major Project, 2024ZD0526900; Tianfu Qingcheng Project‐Tianfu Science and Technology Elite, (No.1358); Xining Joint Logistics Support Center‐Technology Top Talent, to HP; General Hospital of Western Theater Command‐Head Goose Project Training Object, to HP.

## Data Availability

Data available on request from the authors.
